# High prevalence of low bone mineral density but normal trabecular bone score in Norwegian elite Para athletes

**DOI:** 10.3389/fspor.2023.1246828

**Published:** 2023-11-15

**Authors:** Anu E. Koivisto-Mørk, Kathrin Steffen, Trine E. Finnes, Mikkel Pretorius, Hilde Moseby Berge

**Affiliations:** ^1^ Oslo Sports Trauma Research Center, Department of Sports Medicine, Norwegian School of Sport Sciences, Oslo, Norway; ^2^ Department of Sports Medicine, Norwegian Sports Medicine Centre (Idrettens Helsesenter), Oslo, Norway; ^3^ Department of Endocrinology, Morbid Obesity and Preventive Medicine, Oslo University Hospital, Oslo, Norway; ^4^ Section of Endocrinology, Innlandet Hospital Trust, Hamar, Norway; ^5^ Faculty of Medicine, University of Oslo, Oslo, Norway; ^6^ Department of General Practice, Institute of Health and Society, University of Oslo, Oslo, Norway

**Keywords:** osteopenia, osteoporosis, trabecular bone score, disabled athletes, bone stress injury, bone mineral density, Paralympic athlete

## Abstract

**Background:**

Low bone mineral density (BMD) increases the risk of bone stress injuries (BSI) and is one of several clinical concerns in Para athlete sports medicine. However, whether bone microarchitecture is altered in Para athletes is not known.

**Objective:**

We aimed to investigate BMD, bone microarchitecture and incidence of bone stress injuries in Norwegian elite Para athletes.

**Design:**

In this cross-sectional study in Para athletes, Dual energy x-ray absorptiometry (iDXA, Lunar, GE Health Care) derived areal BMD, trabecular bone score (TBS), a surrogate marker for bone microarchitecture, and body composition (body weight (BW), lean body mass (LBM), fat mass (FM), fat percentage) were investigated and compared between ambulant and non-ambulant athletes. Also, the association between BMD, TBS and body composition variables was investigated. Incidence of BSI was assessed with a questionnaire and confirmed by a sports physician in a clinical interview. BMD Z-score <−1 was defined as low and ≤−2 as osteoporotic. TBS ≥ 1.31 was normal, 1.23–1.31 intermediate and <1.23 low.

**Results:**

Among 38 athletes (26 ± 6 yrs, 14 females), BMD Z-score was low in 19 athletes, and osteoporotic in 11 athletes' lumbar spine (LS) or femoral neck (FN). BMD was lower in non-ambulant vs. ambulant athletes both in LS (1.13 ± 0.19 vs. 1.25 ± 0.14 g/cm^2^, *p* = 0.030) and FN (0.90 ± 0.15 vs. 1.07 ± 0.16 g/cm^2^, *p* = 0.003). TBS was normal for all athletes. BMD Z-score in LS was positively associated with TBS (*r* = 0.408, *p* = 0.013), body weight (*r* = 0.326, *p* = 0.046) and lean body mass (*r* = 0.414, *p* = 0.010), but not with fat mass or fat percentage. None of the athletes reported any BSI.

**Conclusions:**

Half of the Norwegian elite Para athletes had low BMD, and 29% had BMD Z-score <−2 suggesting osteoporosis. Non-ambulant athletes were more prone to low BMD than ambulant athletes. However, despite high prevalence of low BMD, TBS was normal in all athletes, and BSI was absent in this young population.

## 1. Introduction

Low bone mineral density with an increased risk of bone stress injury (BSI) is one of several clinical concerns in Paralympic sports medicine (1). Identifying athletes with low BMD is necessary for targeted preventive measures (2).

Athletes' BMD is expected to be 10%–15% higher than age-matched non-athletes (3), but differs between sports (4, 5). Also, in athletes with physical impairment (Para athletes), BMD is supposed to vary by type of physical impairment, ambulatory status and to be site specific (6, 7). There is evidence of a protective effect of exercise to maintain BMD in Para athletes, however, only above the level of injury (6, 8, 9).

Following spinal cord injury (SCI) the rapid decline in BMD (up to 50%) (10) seams to reach a new plateau within 5 years after injury (11, 12). Thus, non-ambulant athletes with congenital (e.g., spina bifida) or long-standing SCI might be more prone for low BMD and BSI than other Para athletes. Also, lean body mass (LBM) in total body declines after SCI (13). Yet, LBM above the level of injury is often maintained or even elevated in non-ambulant Para athletes (14) and is positively associated with BMD, at least in healthy adolescents (15), reflecting the osteogenic effect of exercise-induced muscle-tendon pull, and perhaps the compressive strain to bone induced by resistance exercise (16).

BSI occurs following cyclic overload to bone, leading to microtrauma that may progress to stress reaction. In US elite Para athletes, nearly 9% reported a BSI, of whom more than half had low BMD (17). Such microtrauma is often seen in athletes with low energy availability (LEA) (18, 19). However, while low BMD and BSI are closely associated with low body weight, fat mass (FM) and LEA in able-bodied athletes (20, 21), it is still unclear whether low BMD in Para athletes is due to LEA or rather a consequence of the impairment and unloading (22, 23).

Bone quality beyond BMD has gained interest in athletic populations (19). Bone microarchitecture, measured indirectly with trabecular bone score (TBS) in lumbar spine is an emerging method in the contemporary assessment of bone quality (24). Reduced TBS and BMD in spine are associated with BSI and are surrogate markers of LEA, at least in able-bodied athletes (25). However, the value of TBS in the Para athletes has not been investigated.

Thus, the aim of this study was to describe areal BMD, estimated bone microarchitecture (TBS) and BSI incidence and body composition (LBM, FM) in young ambulant and non-ambulant Norwegian Para athletes, and to investigate associations between BMD in lumbar spine and femoral neck with these variables.

## 2. Materials and methods

### 2.1. Study design

The current study is part of the ongoing clinical pre-participation health evaluation and monitoring program of Norwegian Olympic and Paralympic candidate athletes as described by Clarsen et al. (26). In total, 40 elite athletes, as defined by McKay et al. (27), all candidates for the upcoming Paralympic Games from summer and winter sports were invited to this study. Data on athleteś bone health and body composition were collected at the Norwegian Olympic Sports Centre, Oslo, Norway between August 2020 and May 2022. Informed written consent was obtained from all athletes before the start of the health monitoring program.

#### 2.1.1. Body composition and bone mineral density

All DXA scans were performed after an over-night fast and participants were instructed to avoid rigorous training 24 h prior to the scan and to ensure good hydration status. Any pieces of metal were marked as artefacts manually in DXA post-analysis as instructed by the producer. Prior to DXA scans body weight and height (lying height for non-ambulant athletes) were measured in minimal clothing without prosthesis (Seca portable stadiometer 213, Seca scale 876, or Seca wheelchair scale 664, Birmingham, UK). Body composition (lean body mass (LBM), fat mass (FM)) and bone mineral density (BMD) was measured with dual-energy x-ray absorptiometry (DXA) (Lunar iDXA, GE Health Care, Madison, USA) following the best practice protocol for able-bodied subjects (28). All scans were performed at the same institution and analyzed by the same technician (AEKM) to ensure consistency. BMD value (g/cm^2^) for each athlete was standardized to BMD Z-scores for a reference population accounting for age, sex and ethnicity, using the combined NHANES/Lunar reference database. To define low BMD and osteoporosis, we used the American College of Sports Medicine (ACSM) guidelines (29). They state that BMD Z-score between −1 and −2 is defined as low BMD (osteopenia), while a BMD Z-score <−2 with a secondary clinical risk factor for fracture is defined as osteoporosis (29).

##### 2.1.1.1. Body mass index (BMI)

To define overweight (BMI > 25 kg/m^2^) and underweight (BMI < 18.5 kg/m^2^) WHO's cutoffs were applied for all but those with SCI, where a BMI cutoff (>22 kg/m^2^) for overweight was applied (30).

#### 2.1.2. Trabecular bone score

Trabecular bone score (TBS) is derived from DXA images and based on variations in grayscale pixels and texture to serve as a surrogate marker for bone microarchitecture independent of BMD (31). To extract the TBS values, we utilised TBS software v3.03. in the lumbar spine (L1-L4) after completing TBS iNsight calibration as instructed by the producer (Medimaps group). Normal TBS is defined by the manufacturer as ≥1.31, while intermediate TBS is between 1.23 and 1.31 and low TBS < 1.23, based on meta-analysis evaluating fracture risk by tertiles (32).

#### 2.1.3. Bone stress injury

Bone stress injuries (BSI) were recorded retrospectively with an illness and injury questionnaire (26) and confirmed by a clinical interview by the sports physician.

#### 2.1.4. Statistical analysis

Statistical analyses were performed using IBM SPSS Statistics 24.0. Data are presented as mean ± SD (minimum, maximum) or count (%). The dataset was controlled for signs of non-normal distribution using histograms, QQ-plot and Shapiro–Wilk test. Independent samples Student's *T*-tests were performed to determine differences between ambulant and non-ambulant athletes in the parameters measured. Comparisons between counts (%) in non-ambulant and ambulant categories were analysed with Fisher exact test. One-way ANOVA, with Tukey's post-hoc test for multiple comparisons, was used to assess differences in BMD between the impairment categories. The magnitude of the differences between the groups, i.e., effect size (ES), were determined using Cohen's *d*-test. Correlation between variables was assessed with Pearson's r or Spearman's rho depending on the normality of distribution. Statistical significance was set at *p* < 0.05.

## 3. Results

### 3.1. Description of athletes

Thirty-eight (95%) of the invited Paralympic candidate athletes participated in the study, while two declined due to logistical challenges related to COVID−19 pandemic. The athletes (Table 1) represented 12 different sports: alpine skiing (*n* = 4), athletics (*n* = 3), badminton (*n* = 1), biathlon (*n* = 1), curling (*n* = 2), cycling (*n* = 1), equestrian (*n* = 1), ice hockey (*n* = 15), rowing (*n* = 1), swimming (*n* = 3), table tennis (*n* = 3), and cross-country skiing (*n* = 3). Nearly half of the athletes were non-ambulant, as depicted in Table 1. The athletes were categorised in following impairment categories, including 10 acquired and 28 congenital impairment types: spinal cord injury (*n* = 6), spina bifida (*n* = 11), amputees (*n* = 2), dysmelia (*n* = 5), cerebral paresis (*n* = 5), visual impairment (*n* = 4) and other (*n* = 5).

**Table 1:** Description of the study population.

	All	Ambulant	Non-ambulant
	(*n* = 38)	(*n* = 21)	(*n* = 17)
Gender
Males	24 (63%)	14 (67%)	10 (59%)
Females	14 (37%)	7 (33%)	7 (41%)
Age (yrs)	26 ± 6 (17, 39)	24 ± 6 (17, 38)	27 ± 7 (17, 39)
Height (cm)	168 ± 12 (140, 192)	171 ± 12 (143, 192)	164 ± 12 (140, 183)
Weight (kg)	67.6 ± 12.6 (42.9, 101.4)	71.3 ± 13.1 (43.3, 101.4)	63.0 ± 10.5 (42.9, 89.1)
LBM (kg)	47.9 ± 16.8 (30.7, 70.1)	52.2 ± 11.0 (31.3, 70.1)	42.6 ± 6.6 (30.7, 55.8)
FM (kg)	16.8 ± 7.3 (5.7, 37.2)	15.5 ± 7.1 (5.7, 33.9)	18.3 ± 7.5 (7.4, 37.2)
Fat percentage (%)	25.8 ± 8.8 (7.6, 43.4)	22.8 ± 7.8 (7.6, 42.1)	29.5 ± 8.7 (12.8, 43.4)
Paralympic medalist			
Yes	11 (29%)	6 (28%)	5 (29%)
No	27 (71%)	15 (71%)	12 (71%)
Sports mobility			
Sitting/lying	27 (71%)	10 (48%)	17 (100%)
Standing	11 (29%)	11 (52%)	0 (0%)

LBM, lean body mass; FM, fat mass.

Values are presented as mean ± SD (minimum, maximum) or count (%).

There was considerable variation in body weight and composition between individuals (see Table 1). One athlete was underweight, while 32% of the cohort was by definition overweight. LBM was lower in non-ambulant compared to ambulant athletes for all body parts except for arms (Supplementary Figure S1).

Two athletes lacked data on BMD for femoral neck due to difficulties with positioning on the DXA bench and post-analysis of the scans. None of the athletes had received long-term bone antiresorptive or anabolic bone medications. However, two athletes with SCI had been treated with intravenous bisphosphonates (zoelodronic acid, Aclasta®) at Oslo University Hospital during the previous year of data collection. Both athletes were included in the analysis. Their Z-score in femoral neck was still <−2, and <−1 in lumbar spine. All SCI athletes in the cohort were paraplegic.

#### 3.1.1. Bone mineral density

**Figure 1:**
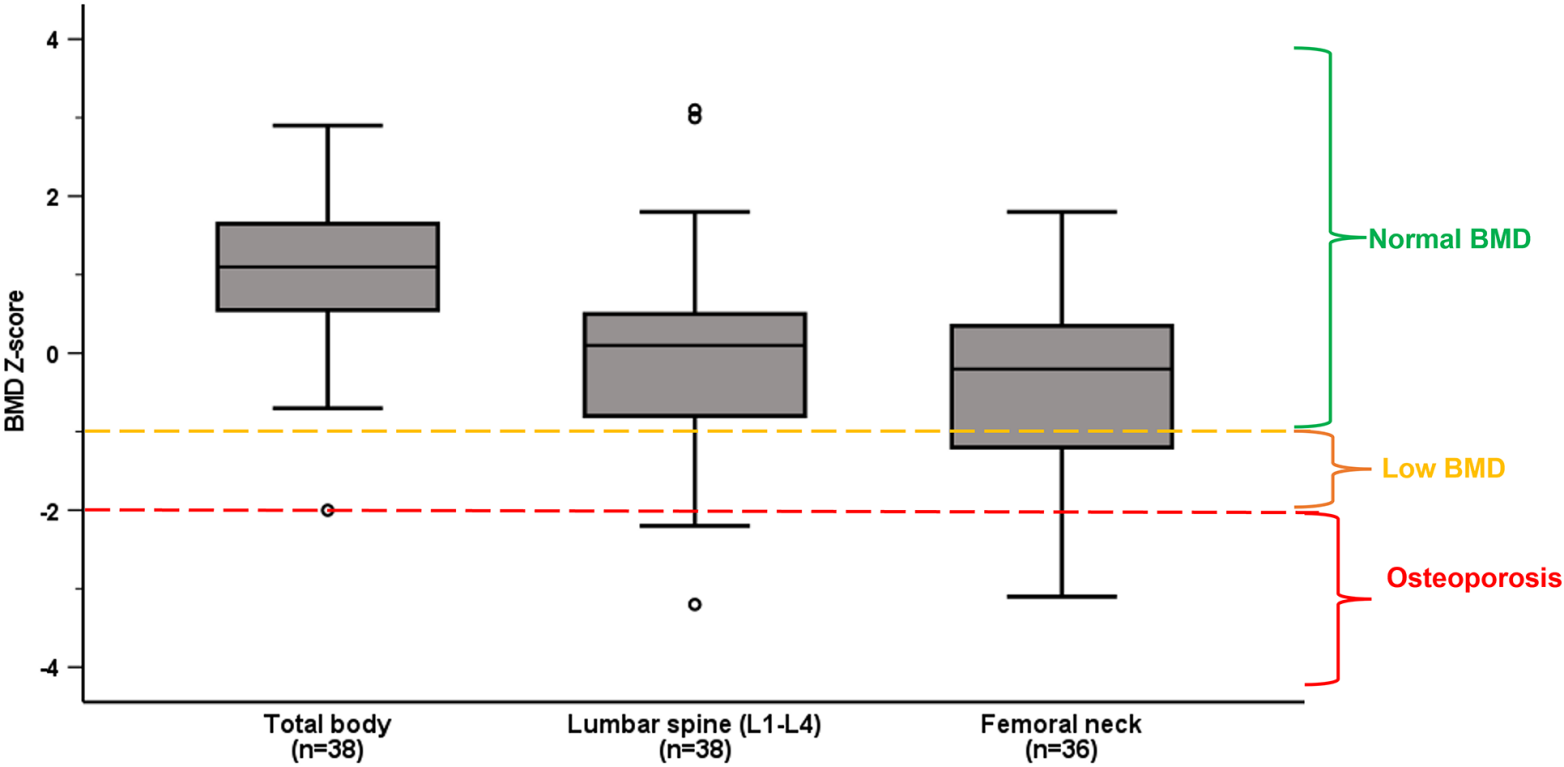
Median and interquartile ranges of BMD Z-score in total body, lumbar spine (L1–L4) and femoral neck in the whole population. Normal BMD, low BMD and osteoporosis are categorized according to ACSM guidelines (29).

Nineteen (50%) of the athletes had low BMD (Z-score <−1), including eleven (29%) with osteoporotic values (BMD Z-score ≤−2) (Figure 1). While 31% (*n* = 11) had low BMD Z-score specifically in femoral neck and 32% (*n* = 12) in at least one of the vertebrae in lumbar spine, we detected four athletes (11%) with low BMD Z-score in both lumbar spine and femoral neck. Only one athlete (3%) had BMD Z-score <−1 in total body.

Low BMD was more prevalent among non-ambulant athletes, with 12 (71%) of them having low BMD compared to seven (33%) of the ambulant athletes (*p* = 0.049). Also, BMD in L3, L4 and L1–L4 was significantly lower in non-ambulant as compared to ambulant athletes (shown in Table 2). There was no difference in prevalence of low BMD between those with congenital vs. acquired impairment (*p* = 0.714), or between male and female Para athletes (*p* = 0.91).

**Table 2:** Bone mineral density (BMD), Z-score and trabecular bone score (TBS) in lumbar spine (LS) vertebra (L1–L4, L1–L4) and femoral neck (FN) in all, ambulant and non-ambulant athletes.

	All	Ambulant	Non-ambulant		
	(*n* = 38)	(*n* = 21)	(*n* = 17)	Mean difference	95% CI
FN					
BMD (g/cm^2^)	0.10 ± 0.18	1.07 ± 0.16	0.90 ± 0.15	−0.17	−0.28 to −0.06
Z-score	−0.4 ± 1.3	0.0 ± 1.1	−1.0 ± 1.1	−1.0	−1.8 to −0.3
LS (L1–L4)					
BMD (g/cm^2^)	1.20 ± 0.18	1.25 ± 0.14	1.13 ± 0.19	−0.12	−0.23 to −0.01
Z-score	−0.1 ± 1.3	0.3 ± 1.0	−0.5 ± 1.5	−0.80	−1.6–0.0
TBS	1.53 ± 0.12	1.55 ± 0.12	1.49 ± 0.10	−0.06	−0.14–0.02
L1					
BMD (g/cm^2^)	1.13 ± 0.16	1.14 ± 0.21	1.12 ± 0.21	−0.02	−0.13–0.09
Z-score	−0.1 ± 1.3	−0.1 ± 0.8	−0.8 ± 1.8	0.0	−0.9–0.9
TBS	1.43 ± 0.16	1.47 ± 0.16	1.37 ± 0.14	−0.09	−0.20–0.05
L2					
BMD (g/cm^2^)	1.22 ± 0.19	1.26 ± 0.16	1.17 ± 0.21	−0.09	−0.13–0.03
Z-score	0.0 ± 1.5	0.3 ± 1.1	−0.3 ± 1.8	−0.6	−1.7–0.5
TBS	1.53 ± 0.13	1.55 ± 0.13	1.51 ± 0.11	−0.05	−0.13–0.04
L3					
BMD (g/cm^2^)	1.25 ± 0.19	1.31 ± 0.16	1.17 ± 0.20	−0.146	−0.26 to −0.03
Z-score	0.2 ± 1.4	0.6 ± 1.1	−0.4 ± 1.6	−1.0	−1.9 to −0.1
TBS	1.57 ± 0.12	1.58 ± 0.13	1.54 ± 0.11	−0.04	−0.12–0.04
L4					
BMD (g/cm^2^)	1.16 ± 0.28	1.26 ± 0.17	1.02 ± 0.34	−0.24	−0.41 to −0.06
Z-score	−0.4 ± 1.6	0.2 ± 1.1	−1.2 ± 1.9	−1.4	−2.4 to −0.4
TBS	1.59 ± 0.12	1.62 ± 0.17	1.56 ± 0.12	−0.06	−0.14–0.02

Values are presented as mean ± SD.

**Figure 2:**
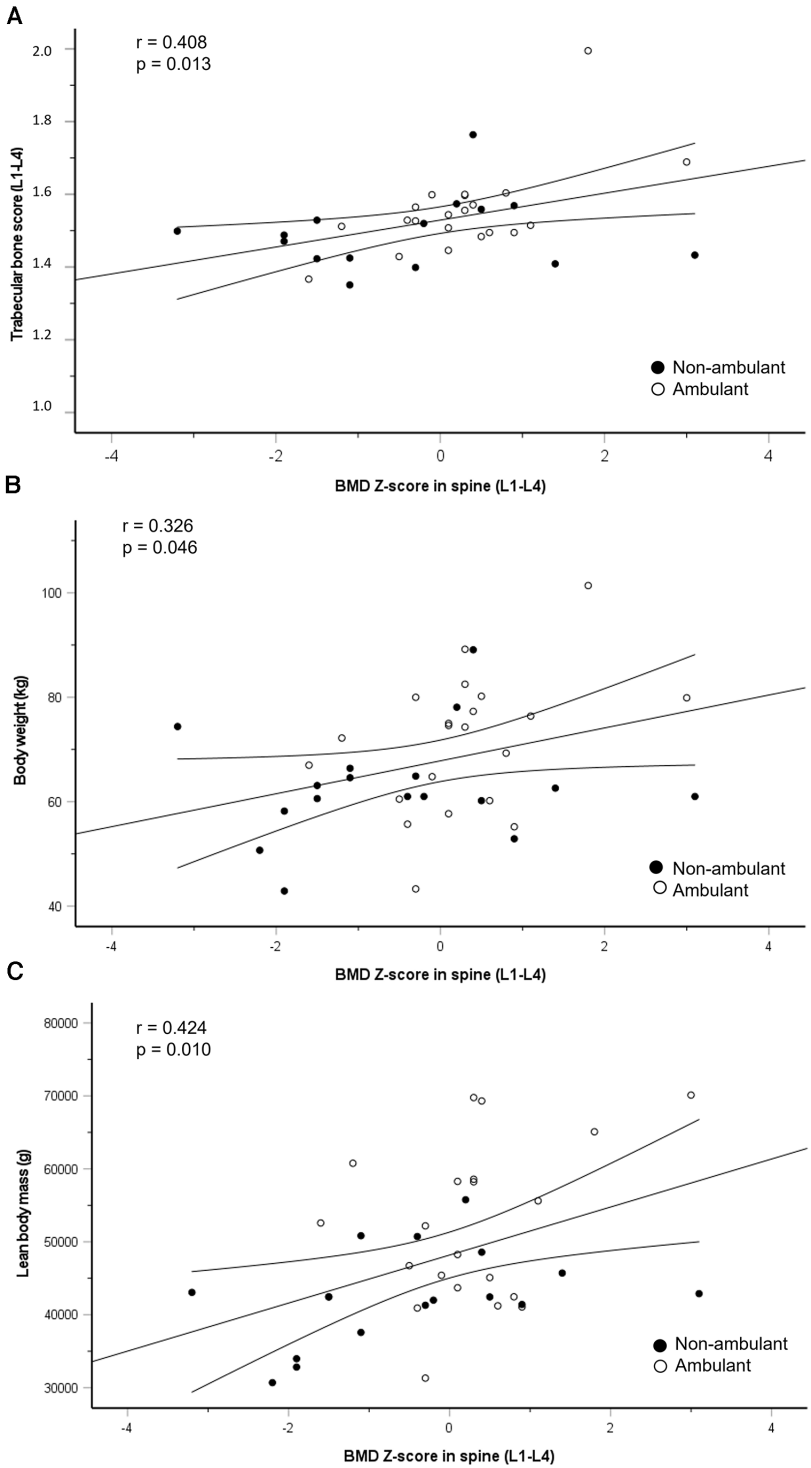
Regression line with 95% confidence intervals for the association between change in BMD Z-score in lumbar spine (L1–L4) and trabecular bone score (L1–L4) (**A**), body weight (**B**) and lean body mass (**C**) closed circles represent non-ambulant athletes and open circles represent ambulant athletes.

BMD in femoral neck was different between the impairment categories, medium ES 0.5, *p* < 0.001. Femoral neck Z-score in SCI was significantly lower than in visually impaired [mean difference (95% CI)] −2.3 (−4.1 to −0.4), *p* = 0.008, and in athletes with dysmelia −2.8 (−4.6 to −1.1), *p* < 0.001 (Figure 2). There were no significant differences in mean BMD Z-score in lumbar spine (L1–L4) between the impairment categories. However, analysis of the lumbar vertebrae BMD individually (L1–L4) showed significant differences between the impairment categories for L3 (ES = 0.3, *p* = 0.05) and L4 (ES = 0.4, *p* = 0.003). Mean BMD (g/cm^2^) was significantly lower in L3 in athletes with spina bifida compared to amputees [mean difference (95% CI)], [−0.44 (−0.860 to −0.021), *p* = 0.035], and in L4 compared to SCI [−0.380 (−0.750 to −0.009), *p* = 0.042], visually impaired [−0.506 (−0.930 to −0.081), *p* = 0.011] and amputees [−0.598 (−1.154 to −0.042), *p* = 0.028].

#### 3.1.2. Trabecular bone score

Mean TBS (L1–L4) in the whole population was 1.529 ± 0.116. All athletes had normal TBS (L1–L4), and no difference was found between ambulant and non-ambulant athletes, contrasting our findings for BMD Z-score, as depicted in Table 2. TBS value analysed for each vertebra individually (L1, L2, L3, L4) showed deviation from normal values only in L1. Five athletes (13%) had intermediate TBS values (1.23–1.31) in L1, while only one athlete had low TBS (<1.23) in L1.

Seven of 38 athletes (18%) had BMD Z-score ≤−2 in L4, of whom 86% (6/7) had spina bifida. However, none of these athletes had compromised mean TBS (L1–L4), as illustrated by a case in Table 3 and Supplementary Figure S2.

**Table 3:** BMD Z-score and trabecular bone score (TBS) in an athlete with spina bifida.

	BMD Z-score	Reference^a^	TBS	Reference^b^
L1	−2.0	≥−1	1.24	≥1.31
L2	−2.9	≥−1	1.56	≥1.31
L3	−3.1	≥−1	1.64	≥1.31
L4	−4.5	≥−1	1.56	≥1.31
L1–L4	−3.2	≥−1	1.50	≥1.31

^a^ BMD Z-score <−1 is by definition low in athletic population.

^b^ TBS ≥ 1.31 is normal, 1.23–1.31 is intermediate and TBS ≤ 1.23 is low.

#### 3.1.3. Association between bone mineral density, trabecular bone score and body composition

**Figure 3:**
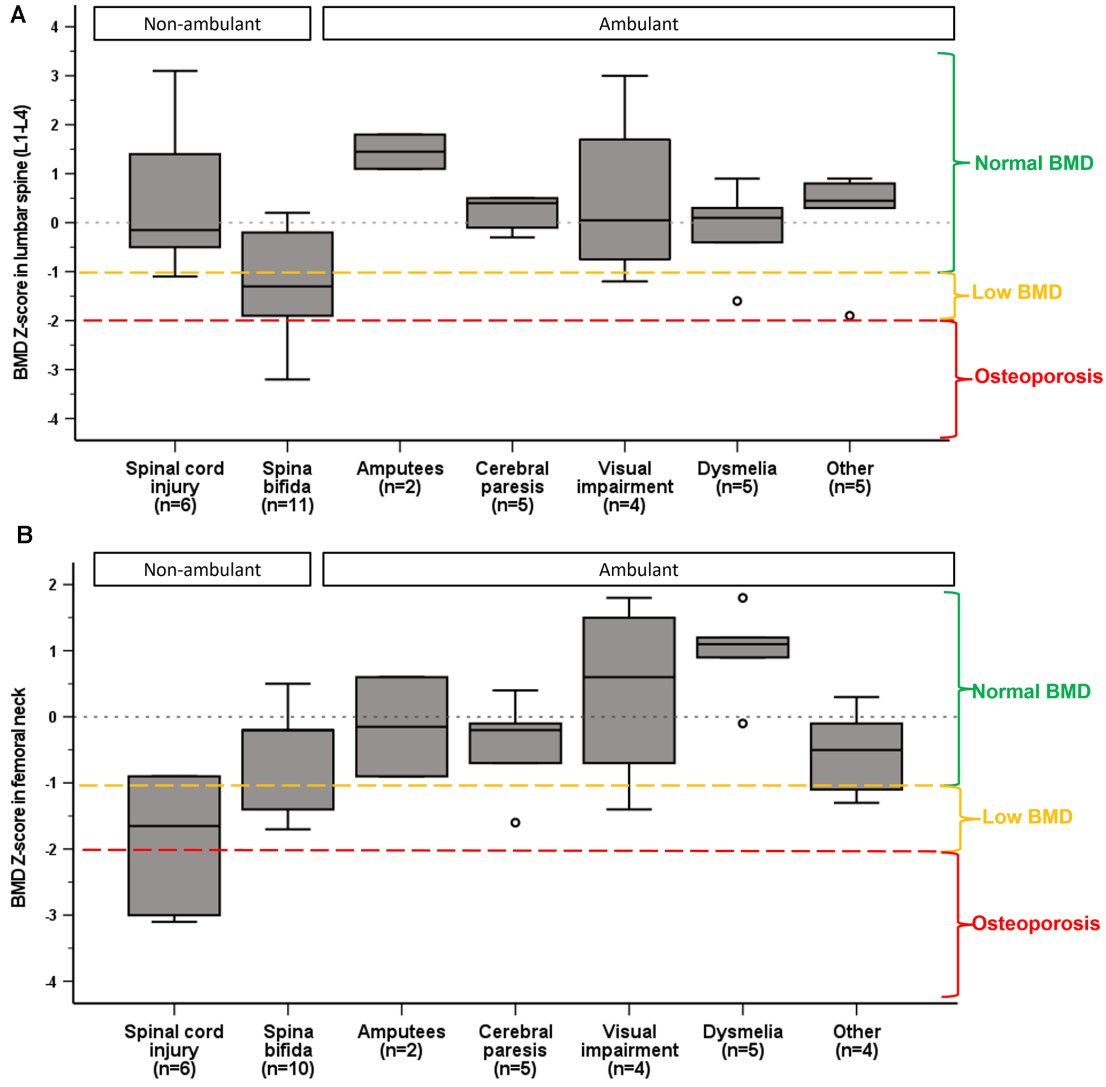
Median and interquartile ranges of BMD Z-score in lumbar spine (**A**) and femoral neck (**B**) by impairment category. Normal BMD, low BMD and osteoporosis are categorized according to ACSM guidelines (29).

BMD Z-score in lumbar spine was positively associated with TBS, body weight and total LBM (Figure 3), but not with total FM or fat percentage.

#### 3.1.4. Bone stress injuries

None of the athletes reported any bone stress injuries or fragility fractures ever.

## 4. Discussion

This study provides novel observations, revealing that half of the Norwegian elite Para athletes had low BMD, including 29% with osteoporotic BMD values. To compare, the prevalence of osteoporosis in able-bodied female athletes has been reported to be between 0% and 13% (29), and ∼5% in Norwegian elite cyclists (33), illustrating the magnitude of this medical concern in Para athletes. Furthermore, we found that non-ambulant athletes were more prone for low BMD. However, despite the high prevalence of low BMD, TBS was normal in all athletes, and BSI was absent in this population. Finally, we found a positive association between BMD Z-score in lumbar spine and TBS, body weight and total LBM, but not with total FM, fat percentage or BSI**.**

### 4.1. Bone mineral density in ambulant vs. non-ambulant athletes

Our finding that BMD was lower in non-ambulant athletes is in line with the existing body of literature for the beneficial effects of weight-bearing physical activity on BMD (16) and the opposing effects of skeletal unloading (34). While non-ambulant athletes' BMD (g/cm^2^) was lower in both lumbar spine and femoral neck, BMD Z-score in lumbar spine was similar between the non-ambulant and ambulant athletes. This may suggest regional differences in BMD. Previous studies have reported that the osteogenic effects of mechanical loading are site-specific (8, 14). For instance, BMD in loaded areas (e.g., forearms) was higher in non-ambulant than ambulant athletes (8) and able-bodied non-athletes (14). The observed partial preservation of BMD in spine might be due to sitting position in the wheelchair (lumbar spine compression) in combination with upper body sporting activity-induced mechanical load (35), or perhaps reflect the absence of low energy availability (18), while persistent deficits in the lower extremities are more likely a result of reduced weight-bearing.

Moreover, we did not detect any difference in BMD in athletes with congenital vs. acquired injury, as might be anticipated because of the difference in time spent in a wheelchair (unloading), including the precious adolescent years of rapid bone mass accrual (36). Indeed, Miyahara et al. found that BMD in trunk, legs and whole body were negatively associated with time since injury in wheelchair athletes (9). Perhaps the timeslot for rapid BMD decline after SCI (11) was already surpassed in our cohort, categorising all SCI athletes with long-standing physical impairment, and accordingly erasing the gap between acquired vs. congenital injury.

Of note, potential artefacts in DXA scans in the spine in individuals with SCI due to degenerative joint disease that may produce falsely high BMD values (37) was absent in our cohort.

### 4.2. Associations between bone mineral density and body composition

Furthermore, we found that LBM was significantly different between ambulant and non-ambulant athletes and LBM was positively associated with BMD Z-score, suggesting that athletes with higher LBM also had stronger bones, analogue to existing literature (15). This may reflect the impairment-dictated proportion of body that can engage in activity leading to muscular adaptations (e.g., hypertrophy) and concomitantly stimulating BMD by muscle-tendon pull. Indeed, the observed difference in LBM between the ambulant and non-ambulant athletes was evident everywhere except for the arms.

Interestingly, we found no association between FM and BMD Z-score. This stands in contrast to findings in able-bodied athletes, showing that low body weight or low body mass index (BMI) and FM are associated with low BMD (21), often as a consequence of LEA (18). Interestingly the association between FM and BMD may also be site-specific, suggesting that BMD in lumbar spine rather than in femoral neck better reflects the systemic energy status, at least in ambulant athletes (38). Low FM *per se* was most likely not a driving factor for low BMD in our cohort, since underweight was nearly absent and 32% of the athletes were overweight.

### 4.3. Bone mineral density by impairment type

We found that BMD Z-score in femoral neck differed by impairment type. Athletes with SCI exhibited lower BMD than athletes with visual impairment and dysmelia. This is not surprising given the well-documented reduction in bone mass following SCI (35), and that visual imparment does not restrict loading of bones, and in theory those athletes should not be exposed to low BMD. Moreover, athletes with dysmelia who use prosthesis, can partially preserve BMD in the lower extremities (39).

Noteworthy, our exploratory analysis of individual vertebra showed that athletes with spina bifida were most susceptible for low BMD in L4 vertebra, accounting for 86% of the cases. This may be explained by the presence of vertebral arch deficits that can falsely lower the lumbar spine BMD (37, 40). Accordingly it has been suggested that BMD should be assessed only in L1 and femoral neck in individuals with spina bifida (40), our findings supporting this notion.

### 4.4. Trabecular bone score

Our study is the first to report TBS values in Para athletes. We found that despite high prevalence of low BMD, mean TBS (L1–L4) was normal in all athletes. Yet, TBS was positively associated with Z-score L1–L4, indicating TBS provides information comparable to BMD. Tenforde et al. (25) showed a similar agreement between TBS and BMD in a large group of 321 able-bodied athletes, with a notion that TBS did not provide added value to estimates of skeletal integrity.

Furthermore while low TBS (<1.310) has been associated with both a history of fracture and the incidence of new fracture in post-menopausal women (31), the cut-off for low TBS in US collegiate athletes to properly predict fracture risk was subsequently higher, 1.419 (25). Utilising that cut-off, four athletes in our study would be at increased risk for BSI.

Perhaps TBS does not properly capture bone microarchitecture in this population with wide range of physical impairments and morphological differences which may affect the bone tissue. Indeed, while normal TBS in our cohort may suggest lower risk for BSI than the observed low BMD alone would imply.more research with TBS in Para population is required before any firm conclusions of its utility can be made.

### 4.5. Bone stress injuries

None of the athletes in our cohort reported any previous BSI ever. Thus, there was no association between low BMD and BSI in our cohort. This contradicts previous studies demonstrating a strong association between BSI and low BMD in Para athletes (17). Among elite US Para athletes 9% reported a BSI, and 12 of those athletes (55%) had low BMD (17). Perhaps, low BMD in this non-weight bearing population does not directly translate to increased risk for BSI, given the definition of low BMD (Z-score <−1) originates from female athletes in weight-bearing sports (41). Importantly, even if the clinically relevant consequences of low BMD (e.g., BSI) were not evident in our cohort, we must address the importance of facilitating peak bone mass accrual in these young athletes during their sporting careers, given low peak bone mass is a major risk factor for development of osteoporosis in later life (42).

### 4.6. Limitations

The heterogeneity of the population is one of the biggest limitations of the study, reducing the power of the statistical analysis. Nonetheless, this study aimed at describing the Norwegian elite Para athlete population as it exists, and therefore included various types of physical impairments. The overall finding (high prevalence of low BMD) may not be directly extrapolated to other Para populations since the diversity in type and severity of physical impairment and ambulatory status may vary between nations. Also, athletes in this cohort were Paralympic candidates, thus similar results may not be anticipated in junior/recruit level athletes. High-resolution peripheral quantitative computed tomography (HRpQCT) was not available for the assessment of bone geometry or structure, which could have provided a more detailed description of the bone microarchitecture, including separate analysis of the trabecular and cortical compartments which may be affected differentially by physical impairment or loading/unloading (43, 44). There is a need to validate TBS against direct bone microarchitecture assessment methods such as HRpQCT in Para athlete population. Finally, we did not consult a radiologist with DXA images, which may be suggested to further improve the quality and precision of DXA imaging of unusual bone segments in Para athletes.

### 4.7. Clinical implications

Non-ambulant athletes should be prioritized for BMD assessments (DXA) to identify those with compromised BMD, even without a BSI. TBS in our cohort may suggest lower risk for BSI than the observed low BMD alone would imply, however, the paucity of research of TBS assessment in Para athletes prevents firm conclusions. BMD in lumbar spine in athletes with spina bifida should be interpreted carefully. The low BMD may illustrate the impairment (hernia) induced anatomical difference rather than a reduction in bone density. Utilizing BMD in L1 and femoral neck might be more correct of the skeletal health, as suggested in non-athletes.

Finally, despite the absence of clinically relevant consequences of low BMD in our population, improving BMD and reducing the risk of BSI should be a prioritized task for the multidisciplinary team of Para athletes.

## 5. Conclusions

In conclusion, this study demonstrates high prevalence of low BMD in Norwegian elite Para athletes. Non-ambulant athletes and especially athletes with SCI were prone to low BMD. Low BMD in lumbar spine in Para athletes with spina bifida needs careful interpretation. Despite low BMD, TBS was normal in all athletes and BSIs were absent in this young Para athlete cohort.

## Data Availability

The original contributions presented in the study are included in the article/supplementary materials, further inquiries can be directed to the corresponding author/s.
