# Supplementary figures and images for: High prevalence of low bone mineral density but normal trabecular bone score in Norwegian elite Para athletes
Source: Front Sports Act Living. 2023 Nov 15;5:1246828. doi: 10.3389/fspor.2023.1246828 (PMC10684761; doi:10.3389/fspor.2023.1246828)

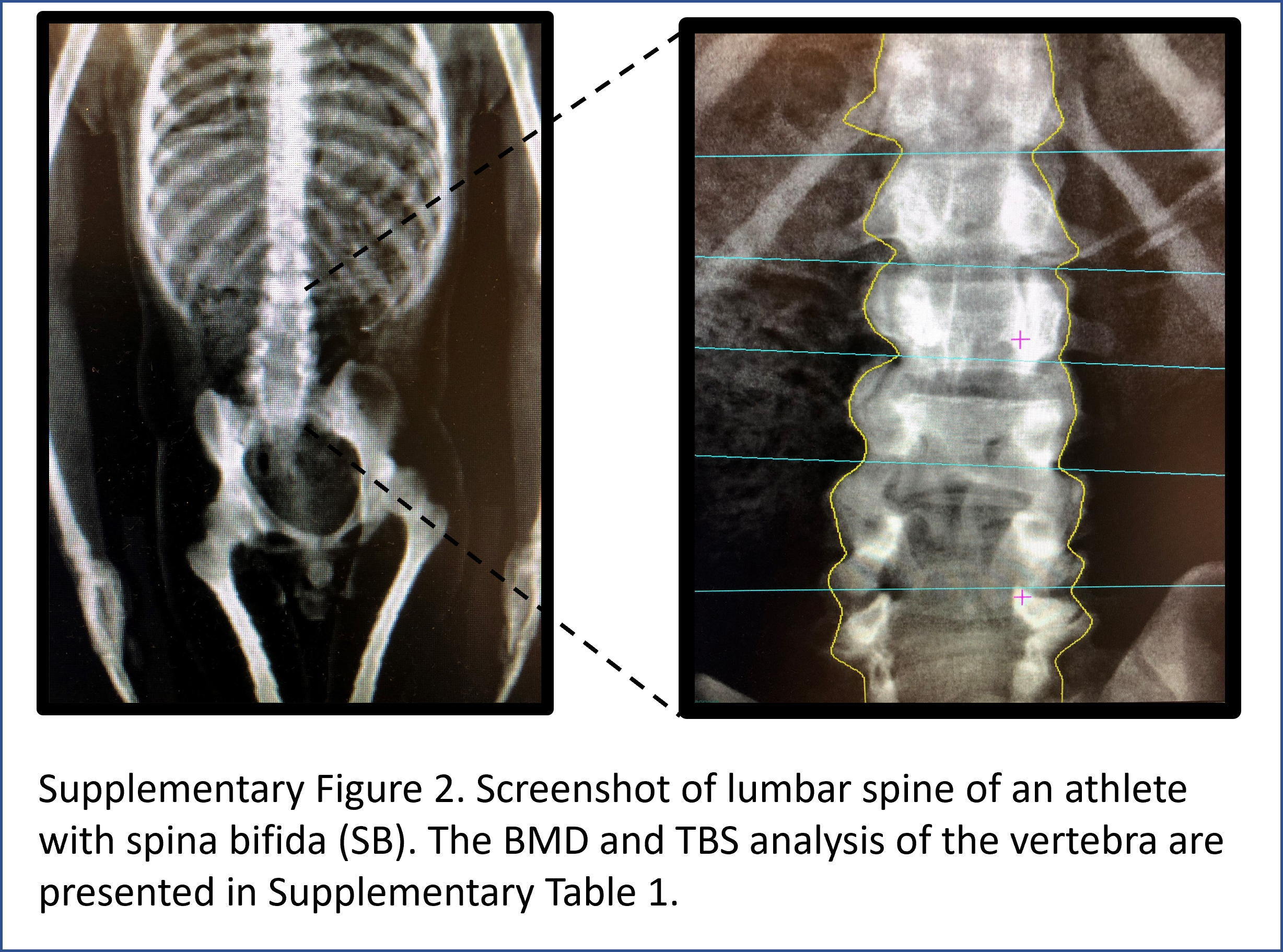

Supplement: Supplementary file 1 [file Image1.png]

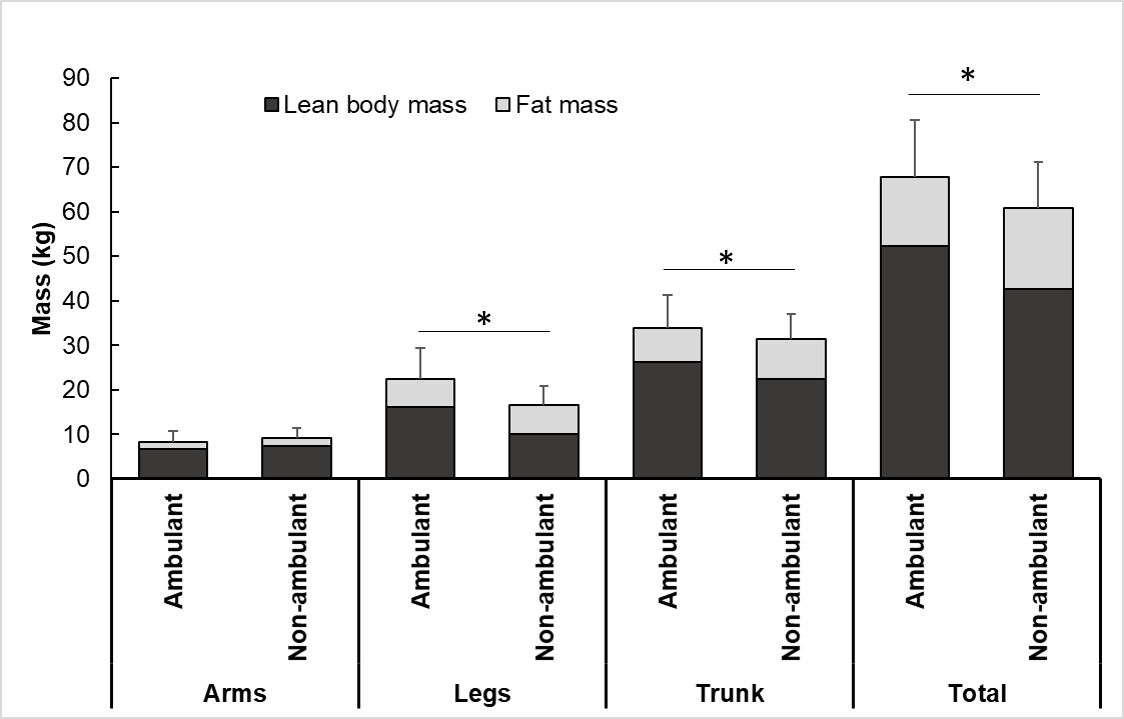

Supplement: Supplementary file 2 [file Image2.tif]
